# Treatment of Mpox with Suspected Tecovirimat Resistance in Immunocompromised Patient, United States, 2022

**DOI:** 10.3201/eid2912.230849

**Published:** 2023-12

**Authors:** Caitlin A. Contag, Leah Mische, Isabel Fong, Abraar Karan, Akanksha Vaidya, David W. McCormick, William Bower, Jill K. Hacker, Kelly Johnson, Perla SanJuan, Linda Crebbin, Caroline Temmins, Harleen Sahni, Yael Bogler, Joseph D. Cooper, Supriya Narasimhan

**Affiliations:** Stanford University School of Medicine, Stanford, California, USA (C.A. Contag, L. Mische, A. Karan);; Santa Clara Valley Medical Center, San Jose, California, USA (I. Fong, C. Temmins, H. Sahni, Y. Bogler, J.D. Cooper, S. Narasimhan);; County of Santa Clara Public Health Department, Santa Clara, California, USA (A. Vaidya, P. SanJuan);; Centers for Disease Control and Prevention. Atlanta, Georgia, USA (D.W. McCormick, W. Bower);; California Department of Public Health. Richmond, California, USA (J.K. Hacker, K. Johnson, L. Crebbin);; University of California, San Francisco, California, USA (K. Johnson, P. SanJuan)

**Keywords:** mpox, monkeypox, monkeypox virus, MPXV, MPX, viruses, severe infection, tecovirimat, tecovirimat resistance, drug resistance, antiviral drug, human immunodeficiency virus, HIV/AIDS, United States

## Abstract

Reports of tecovirimat-resistant mpox have emerged after widespread use of antiviral therapy during the 2022 mpox outbreak. Optimal management of patients with persistent infection with or without suspected resistance is yet to be established. We report a successfully treated case of severe mpox in California, USA, that had suspected tecovirimat resistance.

Tecovirimat is an antiviral drug approved in 2018 by the United States Food and Drug Administration to treat orthopoxvirus infection. Tecovirimat inhibits orthopoxvirus viral protein (VP) 37, which is involved in membrane formation required for egress-competent virions (*1**–**3*). Outside of a clinical trial, the Centers for Disease Control and Prevention recommends tecovirimat only for persons who have (or are at risk for) severe manifestations of mpox (*4*,*5*). 

A low barrier to tecovirimat resistance by mutations in the VP37 protein encoded by the F13L gene of monkeypox virus (MPXV) has been demonstrated through in vitro and animal studies (*6*). Cases of suspected tecovirimat resistance during the ongoing mpox outbreak have occurred in immunocompromised patients receiving prolonged or repeated courses of tecovirimat (*7**,**8*). 

More data are needed to inform the management of severe mpox, including cases of possible resistance. We report a case of a patient who had HIV/AIDS and prolonged severe mpox with F13L gene mutations concerning for possible tecovirimat resistance who was successfully treated with cidofovir, brincidofovir, and vaccinia immune globulin (VIGIV). Written consent was obtained from the patient to share all protected health information and images included in this case report.

## The Study

**Figure 1:**
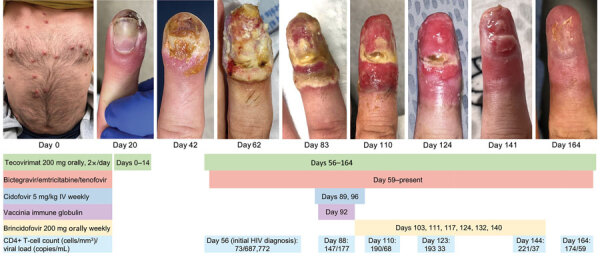
Initial lesions on trunk (day 0, when the patient first sought care at an urgent care clinic) and visual timeline of right index finger lesion of immunocompromised patient with mpox, California, USA, 2022. Treatment received, CD4+ T-cell count, and viral load are indicated below images. IV, intravenous.

A 35-year-old man with no notable history sought care at an urgent care clinic in California, USA, because of painful diffuse skin lesions (Figure 1). He was given a diagnosis of mpox by quantitative PCR from a lesion sample and completed 2 weeks of oral tecovirimat (600 mg 2×/d). No testing for sexually transmitted infections was ordered during his initial examination. He was unable to take the medication with a high-fat meal as recommended. Five weeks after his mpox diagnosis, he sought care for persistent ulceration and swelling of his finger and underwent surgical debridement. Tissue cultures grew methicillin-sensitive *Staphylococcus aureus*, and pathologic analysis showed mixed exuberant inflammation, fibrinopurulent exudates, and pseudoepitheliomatous hyperplasia. Mpox was not suspected and MPXV testing was not obtained. He was prescribed cephalexin for presumed paronychia.

**Figure 2:**
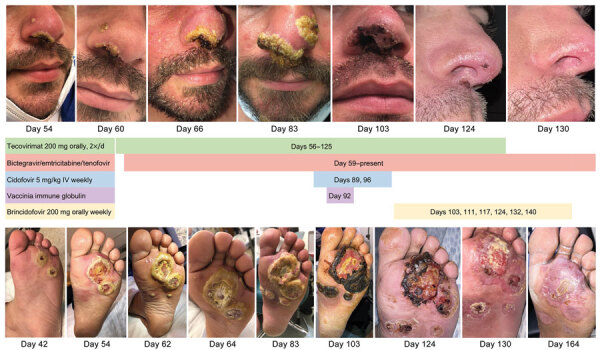
Visual timeline of facial lesions (top) and left plantar lesions (bottom) of immunocompromised patient with mpox, California, USA, 2022. Treatment received is indicated between images. IV, intravenous.

The patient’s lesions continued to worsen, and he came to Santa Clara Valley Medical Center (San Jose, California, USA) ≈8 weeks after his initial mpox diagnosis. He had painful ulcerated lesions on his nose, finger, soles of his feet, anogenital region, and abdomen (Figures 1, 2). The patient had no previous HIV testing; HIV screening obtained at admission was positive with a viral load of 687,772 copies/mL and a CD4+ T-lymphocyte count of 73 cells/mm^3^ (CD4% of 4). His skin lesions were positive for MPXV DNA by quantitative PCR with low cycle threshold (Ct) values, suggesting active infection (nonvariola orthopoxvirus Ct 15.9, MPXV Ct 15.1). Biopsy of an abdominal lesion showed interface dermatitis and marked inflammatory dermal infiltrate.

Oral tecovirimat was again given, and bictegravir, emtricitabine, and tenofovir alafenamide were given within 72 hours of HIV diagnosis. After 1 month of antiretroviral therapy (ART), his viral load decreased to 177 copies/mL, and his CD4+ T-lymphocyte count increased to 147 cells/mm^3^ (CD4% of 8.5). However, despite adherence to both ART and tecovirimat treatment, including taking tecovirimat with high-fat meals, the lesions became progressively larger, more painful, and more deeply ulcerated, and new lesions continued to emerge. (Figures 1, 2). The lesion progression raised concern for possible tecovirimat resistance. Repeat testing of a new chin lesion 4 weeks after restarting tecovirimat confirmed persistence of MPXV with high viral burden (nonvariola orthopoxvirus Ct 20.7, MPXV Ct 17.4). Punch biopsy of the lesion was obtained, and no other underlying etiology was identified.

Given lack of clinical improvement, tecovirimat was continued, and intravenous cidofovir (5 mg/kg/wk for 2 doses), topical 1% cidofovir, and VIGIV (6,000 units/kg) were given (Table). (Topical cidofovir was administered despite the absence of data about the role of topical use because there are no known adverse events associated with topical use and there was a chance that it would benefit the patient.) His lesions became smaller and less painful within days of the first doses of cidofovir and VIGIV. Tecovirimat was continued on the basis of expert guidance from the Centers for Disease Control and Prevention because the drug is well-tolerated and might have synergy with other antiviral drugs (*9**,**10*). The patient was discharged and was receiving ART, oral tecovirimat, and brincidofovir (200 mg orally/wk for 6 doses). Follow-up examinations through day 164 showed continued slow resolution of his lesions (Figures 1, 2).

**Table:** Summary of antiviral therapy received by immunocompromised patient with mpox, California, USA, 2022

Antiviral therapy	Dose	Days administered*
Tecovirimat	200 mg orally twice daily	0–14, 56–125
Cidofovir	5 mg/kg intravenously weekly	89, 96
Brincidofovir	200 mg orally weekly	103, 111, 117, 124, 132, 140
Vaccinia immune globulin	576,000 units intravenously	92

*Day 0 was the date the patient first sought care at an urgent care clinic.

For public health surveillance, MPXV whole-genome sequencing (WGS) was performed on lesion swab samples taken during his initial hospitalization (≈8 weeks after initial diagnosis) and subsequent readmission (≈12 weeks after initial diagnosis); lesion specimens from his initial diagnosis were not available. WGS of the week 8 lesion showed an A290V mutation in the VP37 protein, which has been associated with high-level phenotypic resistance to tecovirimat in orthopoxviruses (*3*,*11*). WGS of 2 lesions sampled at week 12 showed that the mutation VP37 A290V residue had reverted to A290A and a new mutation, D283G, was present. VP37 D283G has been associated with high-level phenotypic tecovirimat resistance in poxviruses (*3*,*11*). The data suggest that resistance to tecovirimat evolved in this case over time.

## Conclusions

During the ongoing multicountry mpox outbreak, mutations associated with tecovirimat resistance have been identified in immunocompromised patients who had severe or persistent mpox and a high mortality rate (*7**,**8*). It is unknown whether this patient was initially infected with a tecovirimat-resistant strain. However, resistance could have emerged during prolonged infection in this immunocompromised patient, who was receiving extended tecovirimat treatment, because 2 resistance mutations were detected in lesions sampled at different sites and timepoints. Different VP37 resistance mutations have been documented in separate lesions from persons who had severe mpox and underwent prolonged tecovirimat treatment (*12*). In addition, the patient might have had subtherapeutic tecovirimat serum concentrations and subsequent selection for resistance when he took his initial course of tecovirimat without high-fat meals (ideally 600 calories or 25 g of fat) (*13*). Challenges for patients living with advanced HIV infection often include lack of housing (*14*) and access to regular food sources, which can pose additional burdens to the specific dietary requirements for tecovirimat absorption (*4**,**15*).

Both the failure to recognize the lesions as progressive mpox and delayed HIV diagnosis probably contributed to the severity of disease in this patient. Current guidelines advise that screening for sexually transmitted infections and HIV be offered to all sexually active patients who have mpox (https://www.cdc.gov/poxvirus/mpox/clinicians/people-with-HIV.html). Earlier ART initiation would probably have benefited the patient. Clinicians caring for patients who have persistent mpox should evaluate for underlying immunodeficiency, particularly HIV, and consider the possibility of antiviral resistance in cases that fail to respond to standard of care. WGS is helping to illuminate the potential resistance mutations that might occur in mpox cases, as well as describe the genetic differences between strains. However, MPXV WGS data are not approved for clinical use, and there is a paucity of data guiding the clinical application of MPXV sequencing. With the likelihood for continued human-to-human transmission of MPXV, expert consultation should be sought for patients who have nonresolving mpox, and lesion samples should be obtained for genotypic and phenotypic resistance testing to inform future case management.

## References

[1] Duraffour S, Snoeck R, de Vos R, van Den Oord JJ, Crance JM, Garin D, et al. Activity of the anti-orthopoxvirus compound ST-246 against vaccinia, cowpox and camelpox viruses in cell monolayers and organotypic raft cultures. Antivir Ther. 2007;12:1205–16. 10.1177/135965350701200802 18240860

[2] Hoy SM. Tecovirimat: first global approval. Drugs. 2018;78:1377–82. 10.1007/s40265-018-0967-6 30120738

[3] Duraffour S, Lorenzo MM, Zöller G, Topalis D, Grosenbach D, Hruby DE, et al. ST-246 is a key antiviral to inhibit the viral F13L phospholipase, one of the essential proteins for orthopoxvirus wrapping. J Antimicrob Chemother. 2015;70:1367–80. 10.1093/jac/dku545 25630650 PMC7539645

[4] Centers for Disease Control and Prevention. Guidance for tecovirimat use. Updated 2023 Feb 23 [cited 2023 Jun 6]. https://www.cdc.gov/poxvirus/mpox/clinicians/Tecovirimat.html

[5] US National Library of Medicine. Study of tecovirimat for human monkeypox virus. STOMP Trial. Updated 2023 Aug [cited 2023 Oct 11]. https://clinicaltrials.gov/ct2/show/NCT05534984

[6] Smith TG, Gigante CM, Wynn NT, Matheny A, Davidson W, Yang Y, et al. Tecovirimat resistance in Mpox patients, United States, 2022–2023. Emerg Infect Dis. 2023;29: 2426–32. 10.3201/eid2912.231146 PMC10683829 37856204

[7] Food and Drug Administration. Tecovirimat drug approval packet. Virology review. Updated 2018 Mar 13 [cited 2023 Feb 16]. https://www.accessdata.fda.gov/drugsatfda_docs/nda/2018/208627Orig1s000MicroR.pdf

[8] Garrigues JM, Hemarajata P, Karan A, Shah NK, Alarcón J, Marutani AN, et al. Identification of tecovirimat resistance-associated mutations in human monkeypox virus, Los Angeles County. Antimicrob Agents Chemother. 2023;67:e0056823. 10.1128/aac.00568-23 37338408 PMC10353411

[9] Quenelle DC, Prichard MN, Keith KA, Hruby DE, Jordan R, Painter GR, et al. Synergistic efficacy of the combination of ST-246 with CMX001 against orthopoxviruses. Antimicrob Agents Chemother. 2007;51:4118–24. 10.1128/AAC.00762-07 17724153 PMC2151443

[10] Rao AK, Schrodt CA, Minhaj FS, Waltenburg MA, Cash-Goldwasser S, Yu Y, et al. Interim clinical treatment considerations for severe manifestations of mpox—United States, February 2023. MMWR Morb Mortal Wkly Rep. 2023;72:232–43. 10.15585/mmwr.mm7209a4 36862595 PMC9997665

[11] Food and Drug Administration. FDA monkeypox response. Updated 2023 Feb 1 [cited 2023 Feb 14]. https://www.fda.gov/emergency-preparedness-and-response/mcm-issues/fda-monkeyp/ox-response

[12] Mitjà O, Alemany A, Marks M, Lezama Mora JI, Rodríguez-Aldama JC, Torres Silva MS, et al.; SHARE-NET writing group. Mpox in people with advanced HIV infection: a global case series. Lancet. 2023;401:939–49. 10.1016/S0140-6736(23)00273-8 36828001

[13] Food and Drug Administration. Tecovirimat package insert. Updated 2022 May [cited 2023 Feb 28]. https://www.accessdata.fda.gov/drugsatfda_docs/label/2022/214518s000lbl.pdf

[14] Aidala AA, Wilson MG, Shubert V, Gogolishvili D, Globerman J, Rueda S, et al. Housing status, medical care, and health outcomes among people living with HIV/AIDS: a systematic review. Am J Public Health. 2016;106:e1–23. 10.2105/AJPH.2015.302905 26562123 PMC4695926

[15] Thakarar K, Morgan JR, Gaeta JM, Hohl C, Drainoni ML. Homelessness, HIV, and incomplete viral suppression. J Health Care Poor Underserved. 2016;27:145–56. 10.1353/hpu.2016.0020 27528794 PMC4982659

